# Effects of Carbon Dots/PVA Film Combined with Radio Frequency Treatment on Storage Quality of Fried Meatballs

**DOI:** 10.3390/foods13223653

**Published:** 2024-11-17

**Authors:** Linlin Zhao, Huinan Jiang, Zhengxuan Han, Wenqin Gu, Xiangren Meng

**Affiliations:** 1 College of Tourism and Culinary Science, Yangzhou University, Yangzhou 225127, China; 008113@yzu.edu.cn (L.Z.); mz120232117@stu.yzu.edu.cn (H.J.); mx120241311@stu.yzu.edu.cn (W.G.); 2 Key Laboratory of Chinese Cuisine Intangible Cultural Heritage Technology Inheritance, Ministry of Culture and Tourism, Yangzhou 225127, China; 3 College of Food Science and Engineering, Yangzhou University, Yangzhou 225127, China; 4 Chinese Cuisine Promotion and Research Base, Yangzhou 225127, China

**Keywords:** carbon dots, active packaging, antimicrobial, antioxidant, sterilization mechanism

## Abstract

The combination of carbon dots/polyvinyl alcohol (CDs/PVA) active film and radio frequency (RF) was performed to improve the storage quality of fried meatball samples. The microbicidal effect of RF, and the antioxidant and antibacterial activities of CDs/PVA film were investigated. The CDs/PVA film can effectively restrain the growth of *B. subtilis*, *S. aureus*, and *E. coli*, and eliminate DPPH and ABTS free radicals. RF exerts inhibitory effects on *C. albicans*, *B. subtilis*, and *E. coli*. For meatball samples, CDs/PVA+RF20 can extend the refrigerated shelf life from 2 w to 6 w. At the sixth week, the total bacterial count (TBC) in CDs/PVA+RF20 group (3.72 log CFU/g) was remarkably lower than those in polyethylene terephthalate (PET) group (7.78 log CFU/g) and CDs/PVA (6.41 log CFU/g) group. CDs/PVA+RF20 can also inhibit the increase in TBARS and POV values. The results manifest the feasibility of CDs/PVA+RF as a novel mild pasteurization or preservation technology.

## 1. Introduction

Prepared dishes generally involve semi-finished and finished foods manufactured with livestock, agricultural, aquatic, and poultry products as raw materials, added with assorted auxiliary materials, and processed through processes including cutting, mixing, shaping, salting, seasoning, and rolling [1]. Prepared dishes are usually stored, circulated, and sold under the conditions of freezing, refrigeration, or normal temperature. In order to prolong shelf life and ensure safety of consumption, the prepared dishes need to be sterilized. Although the conventional thermal sterilization has a good microbicidal effect, it has an adverse influence on the nutritional and sensory quality of prepared dishes. Therefore, the development of innovative mild sterilization technology or preservation technology for prepared dishes is of utmost importance.

Radio frequency (RF) technology has been extensively utilized for the pasteurization of various types of food. Owing to its lower cost, greater penetration depth, volumetric and rapid heating, and better heating uniformity, this technique is considered as a promising alternative for traditional thermal processing [2]. Prior works of research have fully confirmed that RF pasteurization can achieve significant reductions in microorganisms while maintaining better food quality compared to traditional methods [3]. For example, Xu et al. proved that RF 20 min could reduce bacteria in *N. sphaeroides* by 3 log CFU/g, with significantly lower impacts on the rheological properties, water distribution, and texture than high-pressure steam sterilization [4]. Wang et al. examined the impact of RF treatment on the gel characteristics of grass carp myofibrillar protein, and found that RF heating is more advantageous than conventional water bath heating for maintaining the structural characteristics and water distribution of surimi gel [5]. However, due to the low heat pasteurization of RF, when individual RF is not sufficient to meet the requirements of microbiological safety, there is often a need to combine with other techniques to improve the sterilization effect and guarantee the safety of food storage [6].

Active packaging technology can also inhibit the reproduction of microorganisms in food and achieve the purpose of extension of the shelf life [7]. General polymer matrices do not exhibit the UV-blocking property, antioxidant, and antibacterial activities, which are crucial for food preservation. Therefore, it is necessary to change the polymer network by the cross linking with additional components, thereby augmenting its efficacy in food preservation [8]. Due to the rapid advancements of nanotechnology, the construction of multifunctional and efficient active packaging materials by adding functional nanoparticles to polymer matrices has gradually become a hot research topic. Carbon dots (CDs) are a novel type of carbon-based nanomaterials, characterized by their diminutive size (less than 10 nm). It have various advantages, including being synthetic and low cost, and having good biocompatibility, non-toxicity, antibacterial, and antioxidant properties, and so on [9]. Owing to theses excellent properties, CDs have been incorporated into polymer matrixes to produce packaging materials with new functions. For example, Uthirakumar et al. fabricated an UV shielding film made of poly methyl methacrylate and CDs, which can shield more than 99% of UV radiation [10]. Salimi et al. prepared an antibacterial active material made of bacterial nanocellulose and CDs, which can restrain the growth of *Listeria monocytogenes* [11]. The carboxymethyl cellulose/CDs film developed by Riahi et al. showed strong antibacterial and excellent antioxidant properties and could prolong the shelf life of lemons [12]. In summary, CD-based composite films can be used as novel safe and multifunctional materials for food preservation.

The main objective of this study was to develop a novel mild pasteurization technology, that is the combination of CD-based active film and RF pasteurization. Taking fried meatballs as the experimental object, the feasibility of this new technology for preservation of prepared dishes was tested. It is expected that this technology can reduce the microbicidal intensity while ensuring the microbicidal effect, and can effectively maintain the storage quality of the prepared dishes. Herein, we firstly prepared an active film by incorporating CDs into the polyvinyl alcohol (PVA) matrix. The measurements of the CDs/PVA film encompassed its structural properties, mechanical strength, and UV-blocking property, as well as its antibacterial and antioxidant activity. Subsequently, the samples of fried meatball that had undergone various RF treatments were packaged using the CDs/PVA film and stored at 4 °C. For evaluating the effects of the novel mild pasteurization technology on storage quality of meatball samples, changes in microbial level, thiobarbituric acid reactive substances (TBARS), peroxide value (POV), total volatile basic nitrogen (TVB-N), and pH values were analyzed.

## 2. Materials and Methods

### 2.1. Materials

Meatball materials, including pig back fat, pig lean meat, salt, ginger powder, white wine, soy sauce, soybean oil, onion powder, starch, and sugar were provided by Yechun Food Co., Ltd. (Yangzhou, China). PVA powder was procured from Sinopharm Chemical Reagent Co., Ltd. (Shanghai, China). *Candida albicans* (*C. albicans*, ATCC 10231), *Escherichia coli* (*E. coli*, ATCC 43894), *Bacillus subtilis* (*B. subtilis*, ATCC 6633), and *Staphylococcus aureus* (*S. aureus*, ATCC 6538) were bought from the Institute of Microbiology, Chinese Academy of Sciences (Beijing, China). The Na^+^/K^+^-ATP enzyme and AKP enzyme reagent kit were purchased from Nanjing Jiancheng Biotechnology Research Institute (Nanjing, China). CDs were synthesized and characterized in the Institute of Food Resource and Comprehensive Utilization at Jiangnan University (Wuxi, China). The antioxidant and antibacterial activities and the special optical properties of CDs were studied.

### 2.2. Production of CDs/PVA Film

**Figure 1:**
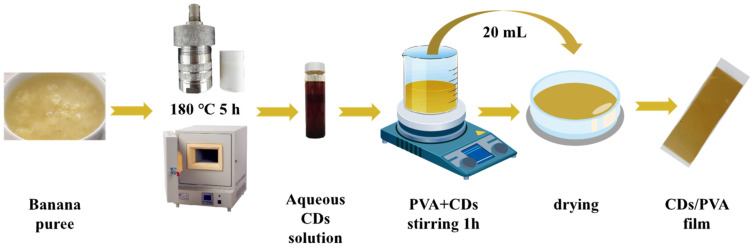
The schematic diagram for the fabrication of CDs/PVA film.

Through the evaluation of the pre-experimental data, the CDs/PVA composite film with 0.5% CDs and 5% PVA was prepared. The film has suitable film color, high transparency, ideal antioxidant and microbicidal effects, and is suitable for the packaging of meatballs. Figure 1 displays the schematic diagram for the production of CDs/PVA composite film. CDs were prepared from banana puree by one-step hydrothermal method (180 °C, 5 h). CDs/PVA composite film was prepared using solution casting method. Aqueous dispersion containing 0.5% CDs and 5% PVA was heated and stirred in 95 °C water until completely dissolved. Then 1% glycerol was alternatively added, and the resulting mixture was agitated for one hour. Finally, twenty mL of film-forming solution was cast onto 10 × 10 cm plastic plate and then dried at 25 ± 2 °C for one day. The preparation process of pure PVA film was the same as the above process, except that CDs were not added. Before further experiments, the prepared 0.5% CDs/PVA film and PVA film were stored at 25 ± 2 °C and 75% RH condition.

### 2.3. Characterization of CDs/PVA Film

The morphology analysis of CDs was analyzed by a transmission electron microscope (TEM, JEM-2100, JEOL, Tokyo, Japan). The particle size of CDs was determined by a particle size analyzer (Nano ZS-90, Malvern, UK). The micro morphological characteristics of CDs/PVA film were analyzed using scanning electron microscopy (SEM, SU8100, Hitachi, Tokyo, Japan). The Fourier-transform infrared (FTIR) spectra of film were gathered through a FTIR spectrometer (Thermo Fisher, Nicolet, iS10, Waltham, MA, USA). The XRD spectra of film were recorded using a X-ray diffractometer (XRD, EDXS, Oxford Instruments, Oxford, UK). The thermal stability of film was tested in a thermogravimetric analysis instrument (TGA2). The UV spectra of film were measured using an UV–vis spectrophotometer (PerkinElmer, Lambda 35, Waltham, MA, USA). The mechanical characteristics of film were assessed by a texture analyzer (TA-XT plus, Stable Micro systems, London, UK). The thickness of film was measured a micrometer (with an accuracy of 0.001 mm). The color parameter of film was detected with a color meter (Konica Minolta, CR-400, Tokyo, Japan).

### 2.4. Functional Properties of CDs/PVA Film

#### 2.4.1. Antioxidant Activities of CDs/PVA Film

The antioxidant activities of the CDs/PVA film were evaluated using the ABTS and DPPH radical scavenging methods. The ABTS analysis was performed as per the method of Roy et al. [13]. In short, 7 mmol of ABTS solution was mixed with 2.45 mmol of potassium sulfate, and the mixture was reacted for 12 h in darkness. Next, 100 mg of CDs/PVA film was mixed with 4 mL of ABTS assay solution. Following a 30 min reaction in darkness, the absorbance was recorded at 734 nm using a spectrophotometer (PerkinElmer, Lambda 35, Waltham, MA, USA). The DPPH analysis was conducted using the methodology outlined by Jamroz et al. [14] with several adjustments. In brief, 100 mg of CDs/PVA film was mixed with 4 mL of 0.1 mmol ethanolic DPPH solution. Following a 30 min reaction in darkness, the absorbance was recorded at 517 nm. The analysis of ABTS and DPPH radical scavenging activity was conducted according to Equation (1):

(1) $$\mathrm{Free}\;\mathrm{radical}\;\mathrm{scavenging}\;\mathrm{activity}\;(\%)=\left(1-A_{i}/A_{0}\right)\times 100\%$$

where *A_i_* and *A*_0_ represent the absorbance values of ABTS or DPPH solution for the test and control solutions, respectively.

#### 2.4.2. Antibacterial Activities of CDs/PVA Film

Agar diffusion method reported by Kousheh et al. [15] with some modifications was used to investigate the antibacterial activity of CDs/PVA film. *B. subtilis*, *S. aureus*, and *E. coli* were activated, resulting in a final concentration of each bacterium at roughly 10^4^–10^5^ colony-forming units (CFU/mL). The 0.50% CDs/PVA solution was loaded onto 6 mm diameter sterilized filter paper. Three kinds of bacterial strains were uniformly applied to the nutrient agar plates, followed by the placement of filter papers onto the surface of the agar. The plates were cultivated at 37 °C for 18 h, then the inhibition zone diameters were recorded.

### 2.5. RF Sterilization Effect and Sterilization Mechanism

#### 2.5.1. Preparation of Bacterial Suspension

The sterilization effect and mechanism of RF treatment on yeast, Gram-positive bacteria, and Gram-negative bacteria were investigated. Activated bacterial suspensions of *E. coli*, *B. subtilis*, and *C. albicans* were centrifuged at a speed of 8000 rpm for 10 min. The precipitates were gathered, rinsed twice, and then suspended with sterile saline solution. The ultimate concentration of each bacteria was estimated to be around 10^7^–10^8^ CFU/mL [16].

#### 2.5.2. RF Sterilization Effect

One hundred g of bacterial suspension was divided into sterile cooking bags and treated with a RF heating device (6 kW, 27 MHz, Model SO6B, Strayfield Co. Ltd., Berkshire, UK). The plate spacing was adjusted to 20 mm, 30 mm, and 40 mm, while the samples underwent treatment for durations of 10 min, 20 min, 30 min, and 40 min, respectively. The decline value of total bacterial count was measured to assess the microbicidal effect of different RF treatments against *E. coli*, *B. subtilis*, and *C. albicans*. In accordance with the method reported by Xu et al. [17] with some modification, the treated bacterial suspensions were diluted 10-fold gradient with sterile saline solution. One mL of dilution was mixed with plate counting agar. After cultivating *E. coli* and *B. subtilis* at 37 ± 1 °C for 48 h, and incubating *C. albicans* at 28 ± 1 °C for 3 d, the total number of colonies was obtained. The sample without RF treatment served as control. We calculated the decline value (*S*) of the total number of bacteria according to Formula (2):

(2) $$S=\log_{10}N/N_{0}$$

where *N*_0_ represents the number of colonies without sterilization, and *N* represents the number of colonies after sterilization.

#### 2.5.3. Sterilization Mechanism of RF Treatment

One hundred g of bacterial suspension was divided into sterile cooking bags and treated with a RF heating apparatus. The plate spacing was adjusted to 20 mm, and the samples were treated for 10 min, 20 min, or 30 min. All samples were placed in the same position of RF heating container for processing.

##### Determination of Relative Conductivity

Changes of relative conductivity were used to reflect changes in bacterial membrane permeability [18]. Activated bacterial suspensions of *E. coli*, *B. subtilis*, and *C. albicans* were centrifuged at a speed of 8000 rpm for 10 min to collect bacterial precipitate. The precipitate was subsequently rinsed twice and re-suspended in sterile 5% glucose solution. The conductivity of resulting suspension, denoted as *L*_1_, was recorded using a conductivity meter (OHAUS, 3100C, Parsippany, NJ, USA). After different RF treatments, the conductivity *L*_2_ was recorded. The bacterial suspension was then heated with boiling water for 10 min and the conductivity *L*_0_ was tested. The relative conductivity was analyzed using Equation (3):

(3) $$\mathrm{Relative}\;\mathrm{conductivity}\;(\%)=\left(L_{2}-L_{1}\right)/L_{0}\times 100$$

##### The Extracellular Protein and Nucleic Acid

The release of protein and nucleic acid into the supernatant can reflect the cell membrane integrity of bacteria [19]. After different RF treatments, the samples were centrifuged at a speed of 8000 rpm for 10 min. Subsequently, the resulting supernatants were collected. The absorbance values at 280 nm and 260 nm were measured using an UV spectrophotometer to reflect the amount of protein and nucleic acid leakage.

##### Determination of Alkaline Phosphatase (AKP)

The leakage of AKP in supernatant can reflect the cell wall permeability [20]. The level of AKP activity was quantified by an AKP Assay Kit (Jiancheng Bioengineering Institute, Nanjing, China). The absorbance value at 660 nm was measured for analysis.

##### Determination of Na^+^/K^+^-ATPase

The impact of RF treatment on Na^+^/K^+^-ATPase was measured using an Na^+^/K^+^-ATPase assay kit (Jiancheng Bioengineering Institute, Nanjing, China). As per the manufacturer’ instructions, the absorbance value at 660 nm was measured for analysis.

### 2.6. Experiment on the Storage of Meatballs

The laboratory conducted the preparation of meatball samples, with each sample having a weight of 40 ± 2 g. The meatballs were subjected to frying in soybean oil at a temperature of 180 ± 5 °C for a duration of 5 min. The CDs/PVA film and PVA film were heat-sealed into small bags with a sealing machine. Some meatballs were packaged directly in CDs/PVA and PVA bags after cooling, named CDs/PVA group and PVA group. The remaining meatballs were sterilized using RF heating apparatus with 20 mm plate spacing. The different groups of meatballs had treatment durations of 10 min, 20 min, and 30 min. An infrared thermal imaging camera (Fluke Ti10 Multi-Purpose Imager, Fluke Corporation, Everett, WA, USA) was used to measure the surface temperatures of meatballs. After cooling, the meatballs were packed with CDs/PVA bags, and named CDs/PVA+RF10 group, CDs/PVA+RF20 group, and CDs/PVA+RF30 group. The packaged samples were placed in a refrigerator at a temperature of 4 °C. At regular intervals of one week, samples of five groups were taken out for testing. TBC, pH, TVB-N, TBARS, and POV were determined to assess the effect of CDs/PVA film combined with RF on maintaining the storage quality of fried meatballs.

#### 2.6.1. Determination of TBC

The determination of TBC value was conducted using the method in the National Standard of the PR China GB/4789.2-2010 [21]. Briefly, a 25 g sample of ground meatball was mixed with 225 mL sterile saline solution and shaken well. Next, 1 mL of the mixture was diluted with 9 mL of sterile saline solution, and then continuously diluted to 10^7^ times. Finally, one mL of diluent was thoroughly coated on a solidified plate count agar. The number of colonies were recorded after incubation at 37 °C for 48 h.

#### 2.6.2. Determination of TVB-N Value

TVB-N value was determined using the method outlined by Wan et al. [22]. Briefly, 2 g of ground meatball sample was added to 100 mL deionized water, blended well, and filtered. A 10 mg/mL MgO suspension was introduced into the filtrate, then distilled for 6 min. The distilled solution was subjected to adsorption using H_3_BO_3_ solution (20 g/L). Finally, the solution was titrated with 0.01 mol/L HCl. The TVB-N value was calculated using Equation (4):

(4) $$TVB-N/(\mathrm{mg}/100\;g)=\frac{\left(V_{1}-V_{2}\right)\times 0.01\times 14}{2\times 5/100}\times 100$$

*V*_1_ and *V*_2_ are the HCl volume consumed in the titration sample and blank sample.

#### 2.6.3. Determination of pH Value

The pH value determination was conducted based on method outlined in the National Standard of the PR China GB/9695.5-2008 [23]. In brief, a 20 g sample of meatballs was minced and evenly mixed with 200 mL deionized water, and then homogenized with a homogenizer for a duration of 10 min. After a resting period of 10 min, the pH value was determined using a pH meter.

#### 2.6.4. Determination of TBARS Value

The measurement of TBARS value was achieved following the method of Wang et al. [24] with several modifications. A total of 5 g of minced meatballs was added to 50 mL of a 7.5% thiobarbituric acid solution. The mixture was then homogenated and filtered. Next, 5 mL of the filtrate was mixed with 5 mL of 0.02 mol/L thiobarbituric acid solution. The resulting solution was subjected to a boiling water bath for a duration of 40 min, and stratified by shaking with chloroform after cooling. The absorbance values at 600 and 532 nm of the supernatant were determined. The TBARS value was analyzed following Equation (5):

(5) $$TBARS/(\mathrm{mgMDA}/\mathrm{kg})=(A_{532}-A_{600})/155\times (1/10)\times 72.6\times 1000$$

#### 2.6.5. Determination of POV

The determination of POV was based on the National Standard of the PR China GB/5009.227-2016 [25]. Ground meatball samples were extracted by petroleum ether. Two gram of extraction products was dissolved with CHCl_3_-CH_3_COOH mixture (30 mL). Then saturated KI (1 mL) solution was introduced and reacted in darkness for 3 min. Subsequently, the mixture was added with 100 mL of deionized water and titrated with Na_2_S_2_O_3_ standard solution (0.01 mol/L). The POV value was calculated by utilizing Equation (6):

(6) $$POV/(g/100\;g)=(V-V_{0})\times 0.01\times 0.1269/2\times 100$$

where *V* and *V*_0_ were the Na_2_S_2_O_3_ standard solution volume consumed for samples for tested and control solutions.

### 2.7. Statistical Analysis

All data from the study were noted as means ± standard deviation (SD) with triplicate measurements. The analysis of the variations among the samples was conducted using IBM SPSS statistics 26 software, the significance level is α = 0.05. Figures were graphed using Originpro 2016 program.

## 3. Results and Discussion

### 3.1. Characterization of the CDs and CDs/PVA Film

**Figure 2:**
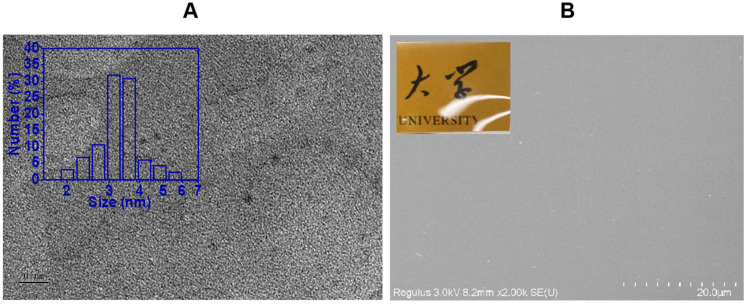
(**A**) TEM image of CDs (inset: particle size distribution of CDs), and (**B**) SEM image of CDs/PVA film’s surface (inset: photographs of CDs/PVA film, the non-English fonts in the picture are Chinese characters, meaning university).

The substrate utilized in this experiment was banana puree for the fabrication of CDs. As shown in Figure 2A, the CDs in the TEM image were monodispersed and nearly spherical without aggregation, and the particle size was less than 10 nm. Figure 2B presents the SEM image of CDs/PVA film’s surface. The CDs/PVA film was observed to be smooth, compact, and without obvious pores and any other irregularities. The inset photograph in Figure 2B shows that the fabricated CDs/PVA film has a high transparency in visible light.

**Figure 3:**
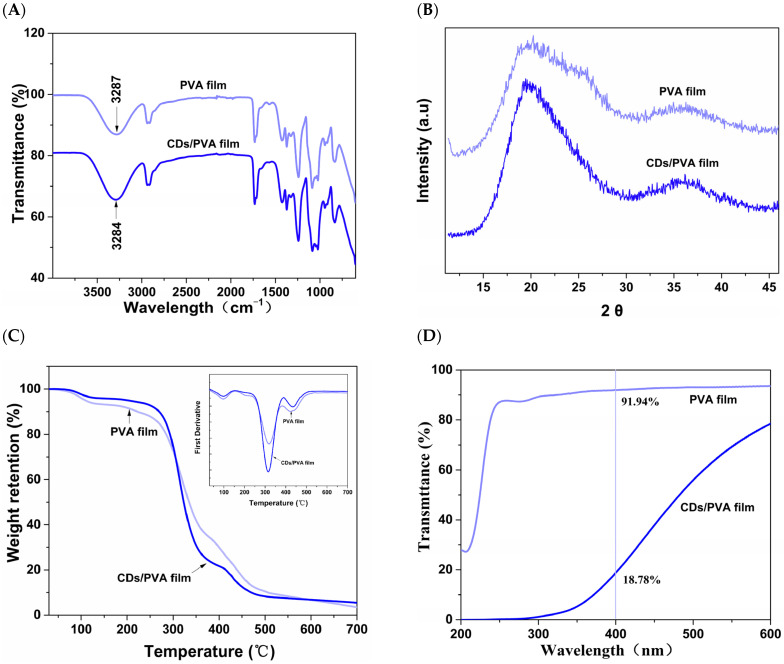
Characterization of CDs/PVA film: (**A**) FTIR spectra, (**B**) XRD spectra, (**C**) TGA curve, (**D**) UV–vis transmittance spectra.

As the FTIR spectra show in Figure 3A, similar absorption peaks were observed in CDs/PVA film and pure PVA film. This similarity can be attribute to the relatively low concentration of CDs present in the film, meaning the absorption peaks of CDs were overlapped and masked by characteristic peaks of pure PVA film. The O-H stretching vibration peak exhibited at 3287 cm^−1^ in pure PVA film slightly shifted to 3284 cm^−1^ in the CDs/PVA film, indicating the formation of new hydrogen bonds between PVA chains and CDs. According to the XRD pattern depicted in Figure 3B, the PVA film exhibited a diffraction peak at a 2θ = 19.6°, reflecting its crystalline characteristics [26]. The diffraction peaks of CDs/PVA film also exhibited similar positions, without new characteristic peaks. The CDs incorporation did not changed the PVA crystal structure, which was consistent with the results of Hu et al. [27].

Figure 3C shows the thermal decomposition process of CDs/PVA film and PVA film. At 150–300 °C, there was a notable upward shift in the TGA curve of CDs/PVA film. At 265 °C, the weight loss of pure PVA film was about 14.41%, and that of CDs/PVA film was only 9.24%. The findings indicate that the incorporation of CDs exerts beneficial impacts for the thermal properties of films made of PVA. At 400–500 °C, the weight losses of the CDs/PVA film was higher, potentially attributed to the breakdown of structures based on carbon. Figure 3D shows the UV-blocking properties of films. Over the entire UV area (200–400 nm), the pure PVA film exhibits a largest UV transmittance, T_400nm_ = 91.94%. With the wavelength ranging from 200 nm to 600 nm, the UV transmittance of CDs/PVA film exhibited a progressive decline. At 400 nm, the UV transmittance of CDs/PVA film fell to 18.78%. This reduction may be attributed to the significant UV absorption properties of CDs, which are capable of altering the energy of light through the absorption of specific characteristic wavelengths [11]. In a similar vein, Patil et al. [28] fabricated a composite film consisting of PVA and CDs. This composite film demonstrated the ability to effectively barrier 100% of UV-C and UV-B, as well as 20–60% of UV-A.

**Figure 4:**
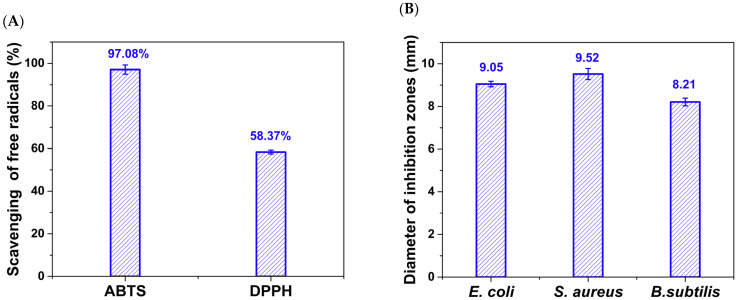
(**A**) Scavenging rate of ABTS and DPPH free radicals, and (**B**) diameter of inhibition zones for *E. coli*, *B. subtilis*, and *S. aureus* of CDs/PVA film.

As shown in Figure 4A, the scavenging rates of CDs/PVA film for ABTS free radical and DPPH free radical were 97.08% and 58.37%, respectively. The DPPH and ABTS free radicals can be reduced and quenched by single electron transfer and/or hydrogen atom transfer from externals molecule. CDs have reductive electron-donating groups (−O−R, −O−C=O, O=C−OH, −OH) on their surface, which can directly react with free radicals to generate more stable products [8]. The ABTS method showed a much higher scavenging rate; the main reason may be the hydrophilicity and good dispersion of CDs in an aqueous medium. In addition, due to the hydrophilicity of PVA, the decomposition rate of CDs/PVA film in ABTS aqueous solution was also higher than that in DPPH ethanol solution. Similar excellent ABTS radical scavenging ability has been observed in CD-added chitosan/gelatin films [29].

Figure 4B displays the antibacterial efficacy of CDs/PVA film-forming solution on *B.subtilis*, *E. coli*, and *S. aureus*. The addition of CDs conferred antibacterial properties to the CDs/PVA film. The CDs/PVA film-forming solution exhibits inhibition zone diameters of 9.05 mm, 9.52 mm, and 8.21 mm against *E. coli*, *S. aureus*, and *B.subtilis*, respectively. The differences in antibacterial properties among bacteria strains could be attributed to disparities in their cellular wall architecture and composition [30].

Table 1 presents the measurements of the thickness, mechanical characteristics, and color parameters for both the CDs/PVA film and the pure PVA film. The analysis reveals that there is no significant difference in thickness between the two films (*p* > 0.05), which may be because of the small particle size of CDs. However, the addition of CDs had a notable impact on the mechanical properties of PVA film. The result indicated an obvious increase in tensile strength from 56.86 MPa to 79.31 MPa (*p* < 0.05), accompanied by a considerable drop in elongation at break from 51.79% to 42.74% (*p* < 0.05). This phenomenon may be due to the large amount of -COOH and -OH groups on the CDs surface, which interact with -OH and -COOH in PVA, resulting in an augmentation of the mechanical strength of the PVA film [31]. Pure PVA film is transparent and has no obvious color. CDs/PVA film showed a notable reduction in luminosity (L* value), as well as a substantial rise in the red value (a* value) and yellow value (b* value) (*p* < 0.05) compared to PVA film, which can attributed to the characteristic brown color of CDs.

### 3.2. Sterilization Effect of RF Treatment

**Figure 5:**
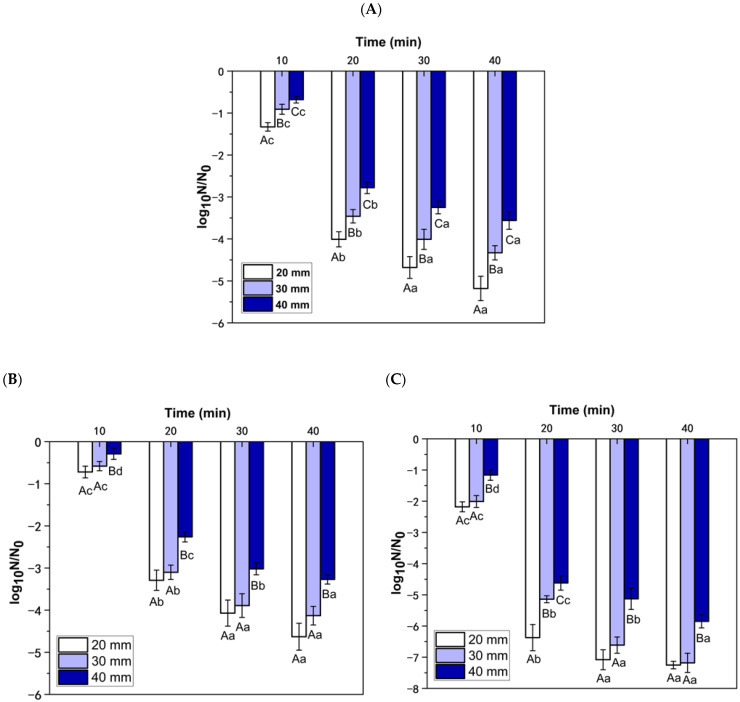
Sterilization effect of RF treatment on (**A**) *E. coli*, (**B**) *B. subtilis*, and (**C**) *C. albicans*. Note: For the same treatment time, different plate spacing, different capital letters represent significant difference (*p* < 0.05). For the same plate spacing, different processing time, different lowercase letters represent significant difference (*p* < 0.05).

Figure 5 lists the sterilization effects of different RF treatments. The lower the log_10_N/N_0_ value, the better the sterilization effect. For *E. coli*, *B. subtilis*, and *C. albicans*, the RF sterilization effect reduced as the plate spacing increased. For the RF instrument used in this test, 20 mm was the minimum plate spacing. Under the same treatment time, the microbicidal effect of 20 mm plate spacing was the best. The reduction in plate spacing resulted in an increase in both the output power and electric field intensity of the RF instrument. Additionally, there was an increase in the energy absorbed by the medium within the electric field, leading to an improved sterilization effect [32]. The study by Lau et al. [33] also proved that when the distance between the upper and lower plate decreased, eggs can absorb more heat energy in the electric field, and the sterilization efficiency increased accordingly. It can also be seen from Figure 5 that under the same plate spacing conditions, the microbicidal effect gradually increased with the increase in treatment time. However, after 20 min of RF treatment, the decrease rate of the colony-forming units of *C. albicans*, *B. subtilis*, and *E. coli* gradually slowed down. RF treatment for 40 min did not bring significant microbicidal advantages, so further research on this time was not conducted in subsequent studies. Under the same conditions, the microbicidal effect of RF against *C. albicans* was better than that of *E. coli* and *B. subtilis*.

### 3.3. Sterilization Mechanism of RF Treatment

**Figure 6:**
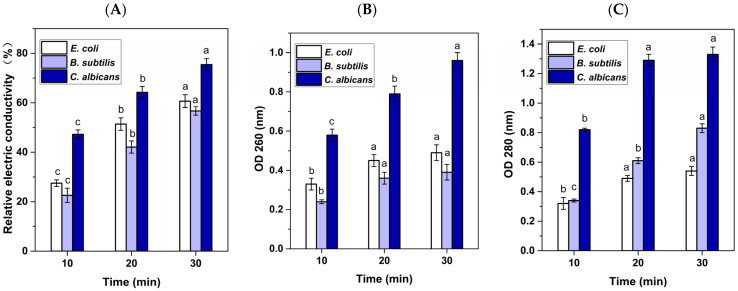
Effects of RF treatment on the (**A**) relative conductivity, (**B**) leakage of nuclear acid, (**C**) leakage of cellular protein of *E. coli*, *B. subtilis*, and *C. albicans.* Note: For the same strain, different lowercase letters represent significant difference (*p* < 0.05).

The integrity of cell membranes is crucial for bacterial growth. The cell membrane of microorganisms is a barrier that allows the passage of small molecules such as K^+^ and Na^+^, which play an important role in maintaining cell membrane biological function, enzyme activity, and normal metabolism of the cell body. The relative conductivity can reflect the changes in membrane permeability after different treatments. As can be seen from Figure 6A, the relative conductivity of *E. coli*, *B. subtilis*, and *C. albicans* show a similar trend, and their values significantly increased as the RF treatment time increased (*p* < 0.05). With RF treatment for 30 min, the relative conductivity of the bacterial suspension was the highest, indicating that RF30 had the greatest impact on cell membrane permeability, leading to a large amount of electrolytes leakage within the cell.

The leakage of bacterial cellular contents is also regarded as an indicator of reduced membrane integrity after treatment with various sterilization techniques [34]. An increase in absorption at 260 nm can reflect the release of intracellular nucleic acids after different RF treatments. According to the results in Figure 6B, the OD_260_ values of bacterial suspension of *E. coli*, *B. subtilis*, and *C. albicans* significantly increased (*p* < 0.05) as the RF treatment time was extended. This increase indicates that membrane integrity was gradually disrupted with the prolonged RF treatment, leading to a notable release of nucleic acids.

The variation in absorbance at 280 nm can reflect the change in the amount of protein discharged into the extracellular milieu and serve as another major indicator of cell lysis and membrane damage [35]. As shown in Figure 6C, the change trend of protein release is consistent with that of nucleic acid, providing additional evidence for the alteration or disruption of membrane integrity in *E. coli*, *B. subtilis*, and *C. albicans* after RF treatment, as well as the increase in microbicidal effect with the increase in treatment time. It is worth noting that for *B. subtilis*, RF 20 min and RF 30 min had no significant difference on the OD_260_ value. For *E. coli* and *C. albicans*, RF 20 min and RF 30 min had no significant difference on OD_280_ value.

**Figure 7:**
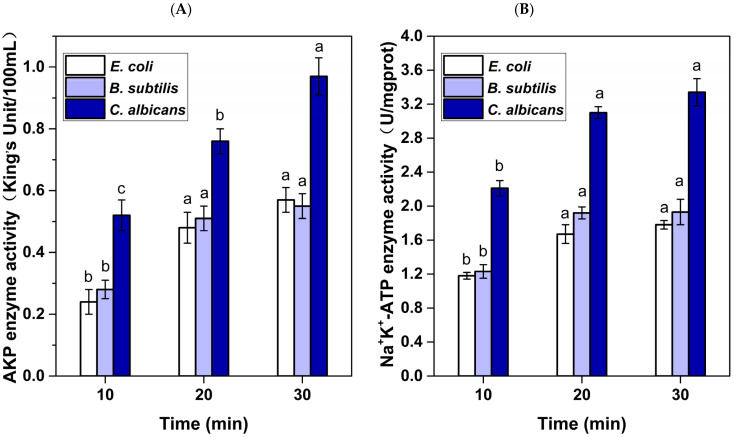
Effects of RF treatment on the (**A**) AKP enzyme activity and (**B**) Na^+^/K^+^-ATP enzyme activity of *E. coli*, *B. subtilis*, and *C. albicans.* Note: For the same strain, different lowercase letters represent significant difference (*p* < 0.05).

The AKP enzyme is a protease that is located in the region between the cell wall and the cell membrane. For normal bacteria, the passage of AKP through the cell wall is highly improbable, so measuring AKP values can reflect changes in cell wall permeability. Figure 7A demonstrates a substantial rise (*p* < 0.05) in the APK value of *C. albicans* as the RF treatment time is extended. However, for *B. subtilis* and *E. coli*, the effects of RF 30 min and RF 20 min on APK values were obviously higher (*p* < 0.05) than that of RF 10 min, but there was no obvious difference between RF 30 min and RF 20 min. These results demonstrate that RF treatment damaged the cell wall, resulting in increased membrane permeability and AKP leakage, and the damage degree was related to RF treatment time.

Na^+^/K^+^-ATPase exists in the cell membrane and controls the transmembrane transport of Na^+^/K^+^ through conformational changes to maintain membrane potential [19], and its activity can reflect changes in membrane permeability. As shown in Figure 7B, there was no significant difference in Na^+^/K^+^-ATPase activity among the samples of *E. coli*, *B. subtilis*, and *C. albicans* that were treated with RF 20 min and RF 30 min. However, the Na^+^/K^+^- ATPase activity in these samples were significantly higher compared to the samples treated with RF 10 min (*p* < 0.05). The above findings demonstrate that the microbicidal mechanism of RF treatment involves disruption of the cell membrane/wall, augmentation of the permeability of the cell membrane/wall, and elevation of the leakage of cell contents.

### 3.4. Temperature Uniformity of RF Treatment

**Figure 8:**
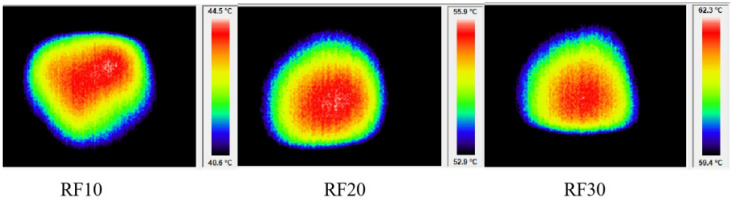
The infrared thermal images of meatball samples after RF treatment.

Figure 8 shows the temperature uniformity observed in the meatball samples after RF treatment. After 10 min, 20 min, and 30 min of RF treatment, the temperature of the samples exhibited an increase to ranges of 40.5–44.5 °C, 52.9–55.9 °C, and 59.4–62.3 °C, respectively. During the thermal treatment, the temperature difference of the sample was well regulated. Notably, no instances of localized heating or excessive heating in specific areas were observed, thereby substantiating the homogeneity of RF heating.

### 3.5. Effect of CDs/PVA Film Combined with RF Treatment on the Storage Quality of Meatballs

In meatballs storage test, we set the storage period as 6 weeks to observe the changes in quality indicators of meatballs, including TBC, TBARS, POV, TVB-N, and pH during a longer storage period. By comparing the changes in the above data among the samples of each group to assess the feasibility of CDs/PVA+RF treatment for preservation of prepared dishes, and explore the mechanism of this new technology delaying the quality deterioration of meatballs.

#### 3.5.1. Microbial Analysis

**Figure 9:**
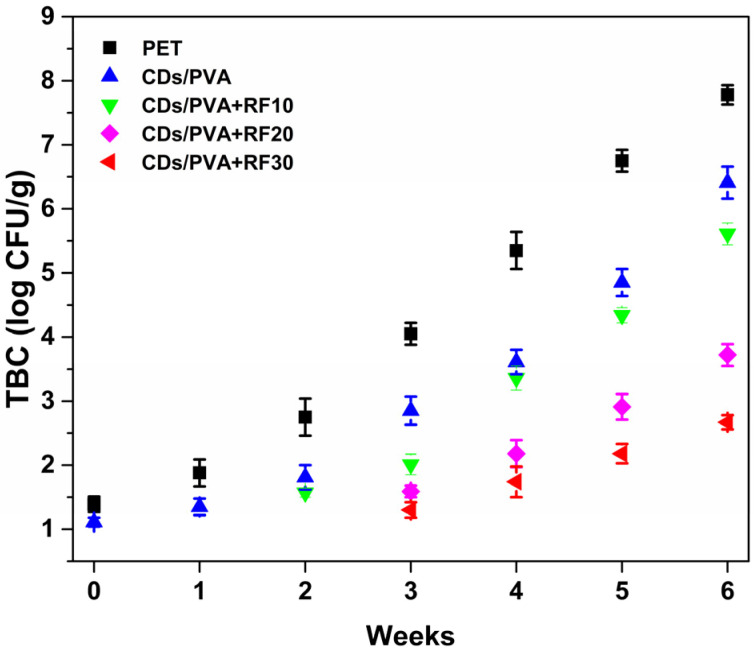
Variation of total bacterial count of meatball samples during storage.

As shown in Figure 9, compared with PET and CDs/PVA groups, CDs/PVA+RF10 groups detected colony growth in the second week, and the CDs/PVA+RF20 group and CDs/PVA+RF30 group detected colony growth in the third week, indicating that RF treatment reduced the microbial population in the samples. Moreover, the microbicidal effects of RF 30 min and RF 20 min were better than RF 10 min. During the storage period, the TBC in all samples showed a gradual increasing trend. The TBC value of CDs/PVA group was significantly lower than that of PET group at the same storage time (*p* < 0.05), indicating that CDs/PVA film could effectively suppress the growth and proliferation of microorganisms during storage. By the third week, the TBC value of the PET group surpassed the safety standard defined by the National Standard of the PR China GB 2726-2016 (10^4^ CFU/g), while the TBC values of the CDs/PVA+RF20 group and the CDs/PVA+RF30 group did not exceed the limit at the sixth week. The above results prove that the CDs/PVA film combined with 20 min or 30 min RF treatment could prolong the shelf life of the sample from the second week to the sixth week.

#### 3.5.2. TVB-N and pH Analysis

**Figure 10:**
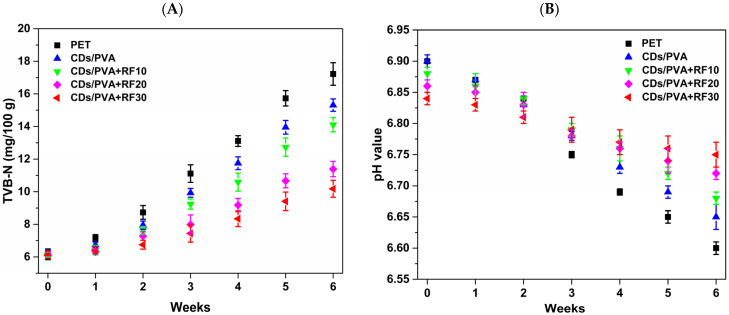
Variation in (**A**) TVB-N and (**B**) pH values of meatball samples during storage.

Figure 10A shows the variation in the TVB-N value. As the storage time extended, the TVB-N levels in all meatballs exhibited a gradual increasing trend, which was due to the accumulation of alkaline nitrogenous substances caused by the proliferation of microorganisms [36]. During storage, the TVB-N levels of CDs/PVA group exhibited a significant decrease compared to PET group. This suggests that the CDs/PVA film could effectively delay the increase in TVB-N levels, which is related to its antibacterial properties. By the sixth week, the TVB-N values of the CDs/PVA+RF20 group and CDs/PVA+RF30 group were 12.87 mg/100 g and 11.18 mg/100 g, respectively. These values were significantly lower than those of the other three groups (*p* < 0.05), and did not exceeded the safety standard specified in National Standard of the PR China GB 2707-2016 (TVB-N ≤ 15 mg/100 g), indicating that CDs/PVA film combined with RF treatment for 20 min or 30 min could effectively reduce the sample decay rate. By the sixth week, the TBC and TVB-N of CDs/PVA+RF20 and CDs/PVA+RF30 samples did not exceed the national standard limit, and it was determined that the shelf life of the meatballs can be extended to 6 weeks.

**Figure 11:**
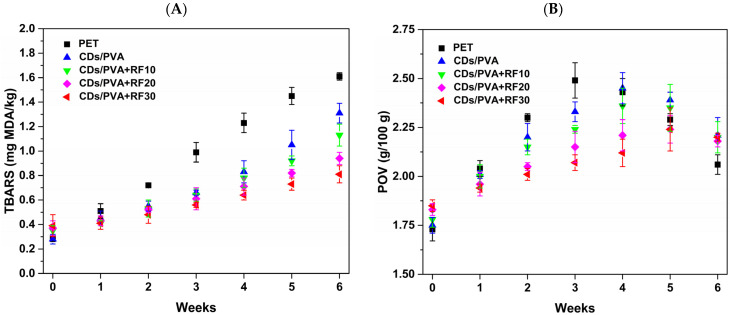
Variation in (**A**) TBARS and (**B**) POV values of meatball samples during storage.

The accumulation of TVB-N is usually accompanied by an increase in pH. However, as microbial activity intensifies, the degree of spoilage increases, producing acidic substances such as lactic acid and acetic acid, ultimately leading to a decrease in pH value. In addition, meat samples are usually accompanied by lipid oxidation during storage, and the acidic products produced also may promote a decline in pH value, resulting in quality deterioration [37]. The intensification of lipid oxidation degree can be reflected by the increase in the TBARS value (Figure 11A). As can be seen from Figure 10B, the pH values of all samples show a downward trend as there is a prolongation of storage time. A similar downward trend was also found in mutton meatball storage test studied by Rubel et al. [38]. After 42 days, the PET group showed the greatest decline. The pH values of the CDs/PVA+RF20 group and CDs/PVA+RF30 group decreased slightly, demonstrating that the CDs/PVA film combined with RF treatment for 20 min or 30 min effectively inhibits the microbial reproduction and lipid oxidation in the samples.

#### 3.5.3. TBARS and POV Analysis

During storage, the changes in TBARS and TVB-N are usually positively correlated with the extension of storage time, and their contents will gradually increase under the action of microorganisms. As shown in Figure 11A, the TBARS values of all samples show an increasing trend as the storage time is prolonged, which may be because the lipid in the meatball samples was easy to be oxidized, and the secondary metabolites gradually accumulated with the deepening of oxidation degree. During weeks 2–6, the TBARS value of the CDs/PVA group was much lower compared to the PET group, indicating that CDs/PVA film could reduce the lipid oxidation degree during storage. This effect was related to the antioxidant properties exhibited by the CDs/PVA film. By the sixth week, the TBARS values of the three CDs/PVA+RF groups exhibited a significant decrease (*p* < 0.05) compared to the CDs/PVA group, which may be due to the multiplication of lipid oxidation and microbial reproduction. As previously stated, the three CDs/PVA+RF groups’ TBC levels were shown to be lower in comparison with the CDs/PVA group alone during the storage period. The CDs/PVA+RF30 group had the lowest TBARS value, followed by the CDs/PVA+RF20 group.

The changes in the POV values of the samples during storage are shown in Figure 11B, and the POV of all samples exhibit a trend of initially increasing and subsequently dropping. The generation and degradation of peroxides are in a state of dynamic equilibrium. During the first phase of oxidation, the degradation rate of peroxides is lower than the generation rate, and POV shows an upward trend [39]. However, when the degradation rate of peroxide is higher than the production rate, POV shows a downward trend. In other words, the decrease in POV does not mean an improvement in lipid oxidation [40]. The POV value of the CDs/PVA group was lower than that of the PET group during weeks 1–3, demonstrating that CDs/PVA film delayed the lipid oxidation of meatballs. During weeks 1–3, POV values of the CDs/PVA+RF20 group and the CDs/PVA+RF30 group were lower than those of the remaining groups. The results suggest that CDs/PVA film combined with RF treatment for 20 min and 30 min has an excellent effect on delaying the oxidative deterioration of meatballs.

According to the above results, due to the relatively short treatment time, to achieve the ideal pasteurization effect and optimum storage quality of the product, CDs/PVA film combined RF heating for 20 min (CDs/PVA+RF20) can be the optimal CDs/PVA+RF treatment.

## 4. Conclusions

A high transparency and active film was fabricated by the incorporation of CDs into a PVA matrix. The interaction between the -OH/-COOH groups in the PVA chain and the -COOH/-OH groups on the surface of CDs forms more hydrogen bonds, improving the mechanical strength and thermal stability of the film. The CDs/PVA film can effectively eliminate DPPH radical and ABTS radical, and the CDs/PVA solution can restrain the growth of *B. subtilis*, *E. coli*, and *S. aureus*. RF treatment exerts inhibitory effects on *E. coli*, *B. subtilis*, and *C. albicans*. Its microbicidal mechanism involves the disruption of cell membrane/wall integrity and permeability, leakage of proteins and nucleic acids. For fried meatball samples, both CDs/PVA+RF20 and CDs/PVA+RF30 can inhibit the increase in TBC and TVB-N during refrigerated storage, extend the shelf life from 2 w to 6 w, and inhibit the increase in TBARS and POV values. The CDs/PVA+RF20 could provide ideal storage quality and relatively short heating time, and is a promising mild pasteurization and preservation technology for prepared dishes.

It is a pity that sensory assessment was not performed in this study. Six weeks of storage should produce a significant change in the sensory characteristics. In future work, we will further study the changes in sensory quality of meatballs during storage, including flavor, taste, texture, and overall acceptability, etc.

## Figures and Tables

**Table 1:** Thickness, mechanical performances, and color parameters of CDs/PVA film.

	Thickness (μm)	Mechanical Performances		Color Parameters		
		Tensile Strength (MPa)	Elongation at Break (%)	L*	a*	b*
PVA	38.75 ± 1.25 ^a^	56.86 ± 1.10 ^b^	51.79 ± 6.95 ^a^	96.77 ± 0.09 ^b^	0.06 ± 0.01 ^b^	1.38 ± 0.06 ^b^
CDs/PVA	40.83 ± 1.44 ^a^	79.31 ± 6.77 ^a^	42.74 ± 4.67 ^b^	75.94 ± 0.21 ^a^	4.44 ± 0.30 ^a^	44.92 ± 0.58 ^a^

Note: For the same column, different lowercase letters represented significant difference (*p* < 0.05).

## Data Availability

The original contributions presented in the study are included in the article, further inquiries can be directed to the corresponding author.
